# Clinical Tests Combined with Color Doppler Versus Color Doppler Alone in Identifying Incompetent Perforator Veins of the Lower Limb: A Prospective Analytical Study

**DOI:** 10.7759/cureus.2026

**Published:** 2018-01-05

**Authors:** Sathasivam Sureshkumar, Narayan Vignesh, J Venkatachalam, Chellappa Vijayakumar, Sundaramurthi Sudharsanan

**Affiliations:** 1 Surgery, Jawaharlal Institute of Postgraduate Medical Education and Research (JIPMER), Puducherry, India.; 2 Preventive and Social Medicine, Jawaharlal Institute of Postgraduate Medical Education and Research (JIPMER), Puducherry, India.

**Keywords:** perforator incompetence, colour doppler, varicose vein, stab ligation

## Abstract

Background

The color Doppler, a better investigation to identify the perforators objectively has replaced the clinical examination for the same. However, this has led to a significant number of negative explorations and cosmetic disfigurement.

Objective

To compare the efficacy of the clinical tests combined with the color Doppler versus color Doppler alone to identify the perforator incompetence during the surgery for primary varicose veins of the lower limb.

Methods

This was a prospective analytical study, including 61 lower limb varicose vein patients who belonged to the Clinical-Etiology-Anatomy-Pathophysiology (CEAP) class four-six, planned for the surgical treatment for perforator incompetence, excluding those requiring additional vascular or nonvascular procedure, recurrent varicose veins and those who had injection sclerotherapy prior to the surgery. The clinical tests, including Trendelenburg’s test, multiple tourniquet tests and, the Fegan’s tests were performed and incompetent perforators were marked on a template as ‘C’ to indicate the clinically positive perforator incompetence. The patients were then examined with the color Doppler ultrasound and the pathological incompetent perforators were marked as ‘D’. The surgical management of the perforator incompetence was done by stab ligation. The incision was made in the color Doppler ‘D’ marked sites as it has been the standard protocol. The number of incompetent perforators identified during the surgical exploration were categorized as ‘D’ positive or ’C’ and ‘D’ positive and were recorded in the specified proforma.

Results

It was found that the mean number of the perforator incompetence identified by the color Doppler alone was 8.2 whereas during the surgery, only a mean of six perforators was identified, leading to 20 unnecessary explorations per 10 patients (8.2 vs. 6; mean difference 2.229; P <0.001). The mean number of the perforator incompetence identified by the color Doppler combined with the clinical tests was 4.5 and during the surgery, a mean of four perforators was identified (4.5 vs. 4; mean difference 0.377; P <0.001). The color Doppler combined with the clinical examination lead to only four unnecessary explorations per 10 patients.

Conclusion

A combination of both the clinical tests and the color Doppler ultrasound has a higher accuracy in detecting perforator incompetence and can reduce the number of negative explorations by the rate of 16 unnecessary explorations per 10 patients.

## Introduction

The varicose vein is the commonest vascular disease and accounts for 10% of the total volume of the cases, in the surgical and vascular out-patient department. Prolonged standing hours, deep venous thrombosis, congenital abnormalities of venous valves are proposed to be the causes of varicose vein diseases; however, in the majority of the cases, the cause remains idiopathic. The clinical tests serve as an important out-patient diagnostic tool to assess the incompetent varicose vein perforators. The clinical examination has a limitation of not identifying accurately all the incompetent perforators and also it requires a reasonable expertise in doing the precise assessment of the vascular abnormality.

The color Doppler is considered as a better investigation to identify the perforators objectively, which excludes the subjective variation among the assessor [1]. An important disadvantage of the color Doppler investigation is that it leads to a significant number of negative explorations as the specificity is low causing a considerable cosmetic disfigurement due to the scar.

The clinical tests can serve as an additive tool with the color Doppler to improve the specificity and can avoid negative exploration [2]. To the best of our knowledge, no study is available in the literature, that compares the efficacy of the clinical tests combined with color Doppler versus color Doppler alone to identify the perforator incompetence during the surgery. Hence, this study was planned to compare the clinical tests combined with the color Doppler Vs color Doppler alone to correctly identify the incompetent perforators in the lower limb varicose vein patients planned for the surgery and to assess the concordance of the clinical tests and the color Doppler to correctly identify the perforator incompetence in the lower limb varicose vein patients planned for the surgery. The study results might help in determining the usefulness of adding the clinical test with color Doppler to avoid negative explorations in the perforator incompetence surgery.

## Materials and methods

This was a prospective analytical study, including all the varicose vein patients planned for the surgical treatment for the perforator incompetence admitted over a period of one year. The Institute Human Ethics Committee approval was obtained for the study. Nature, methodology, and the risks involved in the study were explained to the patient and informed consent was obtained. All the information collected was kept confidential and the patient was given full freedom to withdraw at any point during the study. All provisions of the Declaration of Helsinki were followed in this study.

All the primary varicose vein patients who belong to class four-six of the Clinical-Etiology- Anatomy- Pathophysiology (CEAP) planned for the surgical management for the perforator incompetence in the Department of Surgery were included in the study with the exclusion of those who required additional vascular or nonvascular procedure, recurrent varicose veins and those who had injection sclerotherapy prior to the surgery. The clinical tests, including Trendelenburg’s test, multiple tourniquet tests, and Fegan’s tests were performed by the consultant surgeon and incompetent perforators were marked on a template. The perforators were marked as ‘C’ to indicate clinically positive perforator incompetence.

The patients were then subjected to color Doppler ultrasound examination by the consultant radiologist and the incompetent perforators were marked as ‘D’ to indicate the positive incompetent perforators, identified by the color Doppler. Those perforators with an outward flow of duration of > 500 ms, and with a diameter of > 3.5 mm were considered as the pathological perforators. Three surgery consultants and two radiology consultants performed the clinical tests and the color Doppler investigation respectively. The consultant surgeon who marked the perforators by the clinical tests did not operate on the patients to avoid selection bias. A standard preoperative preparation was done as per the Universal protocol. The patient information including age, gender, symptoms, complications, and the duration of the symptoms was recorded. A standard surgical procedure was carried out for the saphenofemoral junction incompetence. The surgical management of the perforator incompetence was done in the form of stab ligation, where the incision was made in the preoperatively marked site.

All the color Doppler ‘D’ marked sites were explored surgically by mini-phlebectomy incisions for the presence of incompetent perforator as it has been the standard protocol for incompetent perforator surgery. The incompetent perforators were identified and were ligated at the base after the identification of 'T' junction stab phlebotomy technique. The number of incompetent perforators identified during the surgical exploration was categorized as ‘D’ positive or 'C’ and ‘D’ positive and were recorded in the specified data collecting tool. The postoperative care and discharge from hospital were as per the standard Institute's protocol.

Statistical analysis

Assuming that sensitivity of color Doppler with the clinical examination is 80% with a precision of 10% and the desired confidence interval of 95%, the minimal sample required to correctly identify the incompetent perforators was calculated as 61 lower limbs.

The statistical analysis was done by using the Statistical Package for the Social Sciences (SPSS) 19.0 software version for Windows (IBM Corp., Armonk, New York). The categorical variable like gender was expressed as the proportion. The continuous variables such as the total number of incompetent perforators identified by the color Doppler alone and those with a combination of the color Doppler and the clinical examination were expressed as mean with standard deviation. Similarly, the continuous variables like the number of correctly identified incompetent perforators by the color Doppler alone and those with a combination of the color Doppler and the clinical examination were expressed as mean with standard deviation. The difference in the mean of the total number of incompetent perforators identified by the color Doppler and the correct number of the perforators identified by the color Doppler was tested using the Student t-test. Similarly, the difference in the mean of the total number of the incompetent perforators identified by the color Doppler combined with the clinical examination and the correct number of the perforators identified by the color Doppler alone was tested using the Student T-test. A p-value of less than 0.05 was considered statistically significant.

## Results

This was a prospective analytical study, including all varicose vein patients planned for the surgical treatment, for the perforator incompetence admitted over a period of one year in one particular unit in the department of the surgery. Convenient sampling was done and a total of 61 limbs was analyzed for the study. Of the patients, 47 were male and 14 were female.

On comparing the color Doppler positive perforator incompetence and the true positive perforator incompetence (found during the surgical exploration), it was found that the mean number of the perforator incompetence, identified per limb by the color Doppler alone was 8.2, whereas during the surgical exploration a mean of only six perforators was identified, leading to two negative and unnecessary explorations per limb.The mean difference was 2.23 which was statistically significant (Table 1).

**Table 1:** Comparison of the color Doppler positive perforator incompetence with intra-operatively identified perforator incompetence.

Paired Samples Statistics (n=61)			
	Mean	Std. Deviation	p value (Students t test)
Total color Doppler positive perforator incompetence	8.1967	2.2347	0.001
Intra-operatively identified color Doppler positive perforator	5.9672	1.9746	

On comparing the total color Doppler and the clinically positive perforator incompetence with intra-operatively identified incompetent perforators, it was found that the mean number of the perforator incompetence identified by the color Doppler combined with the clinical tests was 4.5 and during the surgical exploration, a mean of four perforators was identified (Table 2). 

**Table 2:** Comparison of the color Doppler and the clinical positive perforator incompetence with intra-operatively identified perforator incompetence.

Paired Samples Statistics (n=61)			
	Mean	Std. Deviation	p-value (Students t-test)
Total positive perforator incompetence to both the clinical examination and the color Doppler together	4.4754	1.3242	.001
Intra-operatively identified color Doppler and the clinical examination positive perforator	4.0984	1.3379	

The color Doppler on an average lead to two unnecessary explorations per patient (20 unnecessary explorations per 10 patients), whereas the color Doppler combined with the clinical examination has led to only four unnecessary explorations per 10 patients.

On analyzing the concordance of the clinical tests and the color Doppler for identification of the perforator incompetence, all the positive perforators identified by the clinical tests were also identified to be positive by the color Doppler examination, indicating 100% concordance between the clinical tests and the color Doppler examination.

## Discussion

The varicose veins are dilated, tortuous veins that occur due to the incompetence of the venous valve or destruction of the valve due to the deep vein thrombosis (DVT). The symptom ranges from dull aching pain in the lower limb, discoloration, heaviness, and cramps to more severe bleeding and non-healing ulcer. The presence of complications like bleeding varicose vein, lipodermatosclerosis, pigmentation, varicose ulcer and the pain interfering with the daily activities are considered as indications for the surgical management.

About 2.5% of the annual healthcare budget in the developed countries like France and Belgium is spent on treating the chronic venous insufficiency. An increasing number of the medical and surgical management is attributed to the increased use of color Doppler ultrasound [3]. The data regarding the economic burden of varicose veins in developing countries are lacking.

There are many clinical tests for detecting incompetence of the perforators. In the cough impulse test, the patient is asked to cough while palpating the Sapheno Femoral Junction (SFJ) or Sapheno Popliteal Junction (SPJ). The tap or percussion test involves tapping the SFJ while palpating the vein for a thrill. These tests tend to miss out a significant number of incompetent perforators (false negative in 36% SPJs and 28% of incompetent SFJs) [4-5]. The Brodie Trendelenburg test for the perforator incompetence has a better sensitivity of 91% [6]. Venography, an invasive imaging procedure that involves the injection of dye and radiographically imaging its flow was used previously to diagnose the venous disorder. Its use has declined in the last decade because of its invasive nature and complications like post phlebographic syndrome (pain, tenderness, erythema, and thrombosis). Also, it has a poor correlation with the severity of the disease [7].

Ambulatory venous pressure monitoring, once considered the gold standard involves connecting a pressure transducer to a cannula introduced into a vein and the fall of blood pressure with 10 tip toe movements was recorded. It is no longer preferred because of its invasive nature and poor localization of incompetent perforators. Air plethysmography is a non-invasive maneuver that measures the air pressure required to milk and empty the veins of blood. It is limited by its poor ability to localize the incompetence.

Noninvasive imaging techniques such as color Doppler can aid in the diagnosis of the varicosities improving the sensitivity further, however, they seem to have reduced the performance of the clinical examination. Duplex scanning is an investigation with high repeatability and non-invasive nature. It is performed with the limb in the dependent position after Valsalva maneuver. It is extremely specific (nearly 100%), but the sensitivity is low [8]. The color Doppler ultrasonography, on the other hand, is highly sensitive but the specificity is low (40%) [9].

Thus a combination of the color Doppler and duplex scanning may be used. But studies have shown that the clinical examination has a high specificity almost equal to that of the Duplex scanning and can reduce the load on vascular labs [10].

The symptomatic patients with no evidence of complications related to varicose veins are usually given a course of conservative management, which includes, lifestyle modification, crepe bandage or graded compression stockings along with limb elevation. However, the considerable number of the patients require the surgical management for a lasting symptomatic cure. The Radiofrequency ablation uses a bipolar catheter to obliterate the vein. Its main advantage is that it has lesser postoperative pain and recovery time. It has the disadvantage of higher cost involved and expertise involved in performing the endovascular procedure in addition to having the highest incidence of the deep venous thrombosis (DVT) (16%) [11]. It is not suitable for the obese patients due to technical difficulty in cannulating the vein.

Endovenous laser treatment works by causing heat injury to vein wall leading to adhesion followed by occlusion of the vein. It has a faster recovery time, and bruising post procedure does not last for more than four weeks. The failure rate for laser ablative procedure increases with increase in the body mass index (BMI) and creation of accidental arteriovenous fistulas have also been reported. Sclerotherapy using either liquid or foam has a low cost and complication rate. However, its long-term improvement and effect on cosmesis are not clearly proven for larger varicosities [12].

The conventional surgeries like stripping of the great saphenous vein and removal of superficial varicosity by stab ligation reduce varied and rely on the calf pump for the circulation of the venous blood. It has a more immediate result with fewer recurrences. The risk of DVT developing after the surgical treatment is 2.1% [13].

The color Doppler investigation is not without limitations which include longer duration required for complete assessment of varicosities, difficult interpretation among obese patients, the requirement of radiologist with reasonable expertise in vascular imaging and higher cost. Considering this higher sensitivity of 97%, the color Doppler investigation is considered a better tool compared to the clinical tests in the evaluation of perforator incompetence before the surgery. Marking of the perforators with the help of color Doppler, the day before the surgery is a routine practice in many centers [14].

In a study done at St James's University Hospital in the UK, it was found that the clinical tests like Perthes and Tap test had a higher positive predictive value (0.83 and 0.80 respectively) compared to handheld color Doppler (0.57 in SPJ). A study in Australia showed that the color Doppler ultrasound, when used alone, led to six false positives in 165 limbs whereas the clinical examination had zero. They advocated the use of the clinical examination alone but this would have resulted in a lesser detection rate of 72% [15].

The present study demonstrated that the color Doppler combined with the clinical examination would combine the high sensitivity of the color Doppler and higher positive predictive value of the clinical examination. The color Doppler examination, when used alone, would have resulted in an average of two unnecessary explorations per patient (20 unnecessary explorations per 10 patients), whereas the color Doppler combined with the clinical examination on an average has led to only four unnecessary explorations per 10 patients.

Though the endoscopic surgery like the Subfascial Endoscopic Perforator Surgery (SEPS) is being performed for the perforator ligation, open subfacial ligation or stab phlebotomy is still the most commonly performed operations for addressing the perforator incompetence, as the SEPS require endoscopic facility and the expertise for doing the endoscopic surgery. Thus, considering the high sensitivity of the color Doppler leading to unnecessary explorations, the addition of the clinical tests would improve the specificity and thus can reduce the number of negative explorations. This advantage can reduce the duration of the surgery by avoiding needless explorations and hence reduce the cost of the surgical treatment and better cosmetic outcome by avoiding a surgical scar.

Limitations

The study was conducted as a prospective analytical study involving a single group of the patients exploring the entire color Doppler positive marked perforator incompetence sites as it has been the standard practice. A randomized control trial comparing two group of the patients could provide a better insight in this regard.

## Conclusions

The color Doppler combined with the clinical diagnosis can reduce the number of negative explorations by the rate of 16 unnecessary explorations per 10 patients. This finding indicates that a combination of both the clinical tests and the color Doppler ultrasound will have a higher accuracy in detecting perforator incompetence. A combination of the clinical tests and the color Doppler assessment would amalgamate the benefits of both, a higher detection rate and a lower false positive rate, thereby reducing the number of negative explorations and disfigurement post surgery.
